# Mechanisms of transcriptional regulation and prognostic significance of activated leukocyte cell adhesion molecule in cancer

**DOI:** 10.1186/1476-4598-9-266

**Published:** 2010-10-07

**Authors:** Judy A King, Fang Tan, Flaubert Mbeunkui, Zachariah Chambers, Sarah Cantrell, Hairu Chen, Diego Alvarez, Lalita A Shevde, Solomon F Ofori-Acquah

**Affiliations:** 1 Center for Lung Biology, University of South Alabama, 307 N. University Boulevard, Mobile, AL 36688, USA; 2 Center of Excellence in Healthy Communities, University of South Alabama, 307 N. University Boulevard, Mobile, AL 36688, USA; 3 Aflac Cancer Center and Blood Disorders Service, Department of Pediatrics, Emory University School of Medicine, 2015 Uppergate Drive, Atlanta, GA 30322, USA; 4 Mitchell Cancer Institute, University of South Alabama, 1660 Springhill Avenue, Mobile, AL 36604, USA

## Abstract

**Background:**

Activated leukocyte cell adhesion molecule (ALCAM) is implicated in the prognosis of multiple cancers with low level expression associated with metastasis and early death in breast cancer. Despite this significance, mechanisms that regulate ALCAM gene expression and ALCAM's role in adhesion of pre-metastatic circulating tumor cells have not been defined. We studied ALCAM expression in 20 tumor cell lines by real-time PCR, western blot and immunochemistry. Epigenetic alterations of the ALCAM promoter were assessed using methylation-specific PCR and bisulfite sequencing. ALCAM's role in adhesion of tumor cells to the vascular wall was studied in isolated perfused lungs.

**Results:**

A common site for transcription initiation of the ALCAM gene was identified and the ALCAM promoter sequenced. The promoter contains multiple *cis*-active elements including a functional p65 NF-κB motif, and it harbors an extensive array of CpG residues highly methylated exclusively in ALCAM-negative tumor cells. These CpG residues were modestly demethylated after 5-aza-2-deoxycytidine treatment. Restoration of high-level ALCAM expression using an ALCAM cDNA increased clustering of MDA-MB-435 tumor cells perfused through the pulmonary vasculature of ventilated rat lungs. Anti-ALCAM antibodies reduced the number of intravascular tumor cell clusters.

**Conclusion:**

Our data suggests that loss of ALCAM expression, due in part to DNA methylation of extensive segments of the promoter, significantly impairs the ability of circulating tumor cells to adhere to each other, and may therefore promote metastasis. These findings offer insight into the mechanisms for down-regulation of ALCAM gene expression in tumor cells, and for the positive prognostic value of high-level ALCAM in breast cancer.

## Background

ALCAM/CD166 is an immunoglobulin cell adhesion molecule expressed by neuronal, endothelial, hematopoietic and epithelial cells [1-13]. It's up-regulation in cancer was first identified at the RNA level in melanoma cell lines as *memD *[14]. Subsequently, increased ALCAM expression was found in melanoma tumors *in situ *[13,15]. More widespread deregulation of ALCAM expression has since been reported in several other tumors including those of the prostate [16,17], esophagus [18], colon [19], bladder [20] and pancreas [21]. Alterations in ALCAM expression in tumors have recently been reviewed by Ofori-Acquah and King [22].

In a study of primary breast cancer tissues and non-neoplastic mammary tissue from the same mastectomies, we discovered that the level of ALCAM transcripts was lower in breast cancer tissues from patients who had metastases to regional lymph nodes [23], and that primary tumors from patients who died of breast cancer had significantly lower levels of ALCAM transcripts [23]. Subsequent studies showed that patients with the lowest level of ALCAM transcripts develop skeletal metastasis [24], that low ALCAM correlated with an aggressive tumor phenotype and significantly negative correlation between ALCAM expression and tumor diameter and grade [25]. More recently high-level ALCAM in breast cancer tissues has emerged as a predictor of good outcome among patients treated with tamoxifen [26] and adjuvant chemotherapy [27,28].

Tumor cells circulate in blood as single entities and multi-cellular emboli [29], and form secondary colonies in the vascular wall. This mechanism of metastasis is supported by evidence showing that tumor cells perfused in isolated rat lungs attach to the endothelia wall with minimum extravasation, leaving the endothelium-attached cells as the seeds of secondary tumors [30]. Indeed, in primary tumors derived from subcutaneous injection of murine breast carcinoma cells in immunocompromised mice, early metastatic colonies are intravascular in origin [31]. That adhesion molecules tethered on tumor cell surfaces influence their colonization of the lung, and downstream metastatic processes, is supported by the finding that the loss of ALCAM at the cell surface confers a high risk for disease progression and mortality in nodal negative cases of breast cancer [26].

In this study, the ALCAM gene was cloned and functionally characterized in a panel of breast cancer and melanoma tumor cell lines, and the influence of ALCAM on homotypic tumor cell adhesion in the pulmonary vasculature investigated. Our findings provide new mechanistic insights on ALCAM that can be developed further to alter its negative influence in tumor cell progression.

## Results

### ALCAM expression in tumor cells

**Figure 1:**
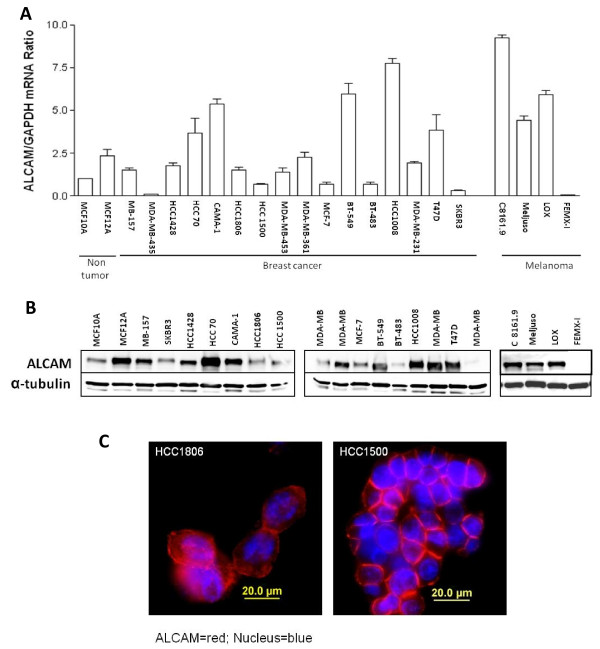
**ALCAM expression in tumor cells**. A) Quantification of ALCAM mRNA by qRT-PCR. Data shown for each cell line is the mean of three analyses each in triplicate. B) Western blot analysis of ALCAM protein in the indicated cells lines. Whole cell lysates (20 μg) were probed with anti-ALCAM antibody, and the filter stripped and re-probed for α-tubulin. C) Immunocytochemical analysis for ALCAM expression and sub-cellular localization in breast cancer cell lines HCC1806 and HCC1500. Cells were stained for ALCAM (red) and nucleus (blue) using the DAPI reagent.

ALCAM mRNA is significantly reduced in primary breast tumors from patients with metastatic disease however the amount of ALCAM in breast cancer cells at metastatic sites remains poorly understood. In this study, ALCAM mRNA in sixteen breast cancer cell lines derived from metastatic breast cancer tumors in the brain, lymph node and the pleural cavity, and primary breast tumors in ductal epithelium were quantified by qRT-PCR. Most cell lines derived from pleural effusions (MB-157, MDA-MB-435, HCC1428, MDA-MB-453, MCF-7, MDA-MB-231 and SK-BR-3) expressed relatively low levels of ALCAM mRNA, while cells originating from the lymph node (HCC70, HCC1008 and BT549) expressed relatively high amounts of ALCAM mRNA (Fig. 1A). ALCAM mRNA was virtually not detectable in MDA-MB-435. Regarding melanoma, ALCAM mRNA was markedly elevated in most of the cell lines (LOX, C8161.9, MelJuso) in agreement with the increased expression in primary tumors (Fig. 1A). Figure 1B shows that ALCAM protein levels determined by western blot analysis showed good correlation with ALCAM mRNA in most tumor cells. Most notably we did not detect ALCAM protein in MDA-MB-435 and FEMX-I tumor cells (Fig. 1B). ALCAM was generally expressed at cell-cell contacts of confluent tumor cell cultures although cytoplasmic localization was also detected (Fig. 1C). These data indicate that ALCAM is variably expressed in breast cancer cell lines, with the lowest level of expression, predominantly in cells derived from distant metastatic sites.

### Structure of the ALCAM promoter and mRNA synthesis at the ALCAM gene locus

The human ALCAM gene is located on the long arm of chromosome 3 [2], therefore we designed primers complimentary to a genomic clone of human chromosome 3 to amplify and sequence 1000 base pairs of a putative ALCAM promoter. Comparing this sequence with entries in the GenBank database identified several reference clones with complete identity to our clone, except for variations of two nucleotides at -154 (A/G) and -618 (C/T) (data not shown). Signal scan analysis revealed that 1 Kb ALCAM promoter contained one copy of a direct repeat, ATTATTATTA sequence, present in the *Drosophila melanogaster *genes encoding transcription factor IIB (TFIIB) and the TATA-box-binding protein (TBP) and no canonical TATA-box.

**Figure 2:**
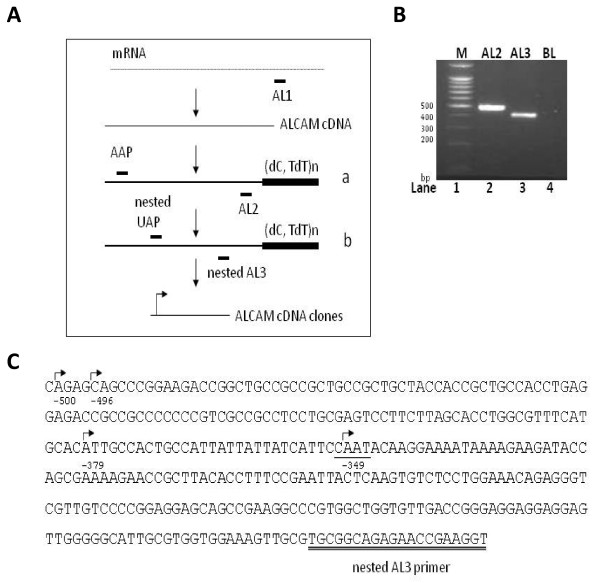
**Transcription initiation sites in the ALCAM gene**. A) Schematic diagram of the 5'RACE approach. Positions of primers AL1, AL2 and AL3 are shown. B) Ethidium bromide stained agarose gel showing DNA marker (lane 1), and products of 5'RACE reaction using primers AAP and AL2 (lane 2), UAP and AL3 (lane 3). Lane 4 is negative PCR control (BL). C) Sequence of a 5' UTR clone of ALCAM showing multiple sites of RNA synthesis (arrows.) The most common site identified is shown underlined. Sequence of AL3 primer is double-underlined.

To identify the site of transcription initiation on the ALCAM gene, complimentary DNA (cDNA) was prepared from non-malignant human cell lines (MCF-12A) and amplified in two sequential PCR analyses using ALCAM-specific reverse primers, and 5'RACE forward primers (Fig. 2A). A single major band of approximately 400 bp was identified in the final nested PCR experiments (Fig. 2B, lane 3). This product was cloned into T-vectors and 20 clones isolated and sequenced. The sequencing data revealed the presence of four putative transcription start sites, located 500, 496, 379 and 349 upstream of the ALCAM translation start site (Fig. 2C). These putative transcriptional start sites had variable agreement with the initiator consensus PyPyA_+1_NT/ApyPy (where Py is pyrimidine), and RNA synthesis usually begins at the adenine (+1). The site at -349 (CCAATA) most closely matched the initiator consensus, with a core sequence [CANT] for strong initiation, with a T at +3 and A at +1, and surrounded by pyrimidines except at position +4. Clones with this site of initiation were the most abundant (65%) identified in our 5'RACE experiments.

### Functional analysis of the ALCAM promoter in tumor cells

**Figure 3:**
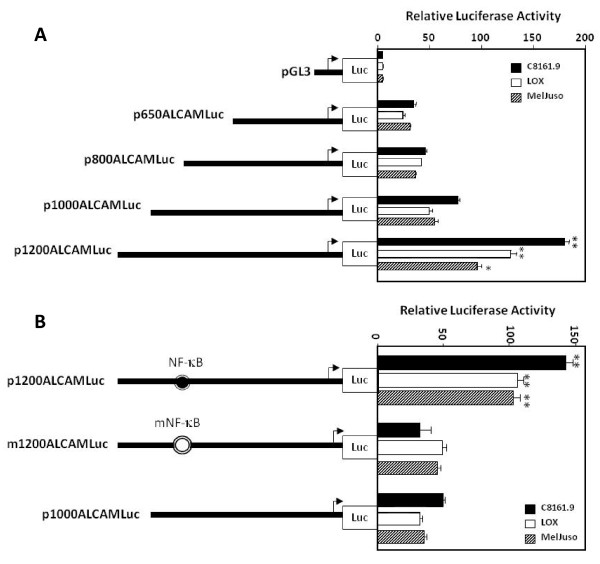
**ALCAM promoter activity in melanoma tumor cells**. A) Schematic diagram of DNA constructs used for reporter gene assays depicting truncations of the ALCAM promoter cloned upstream of a promoter-less luciferase (Luc) gene. Histogram shows the relative luciferase activity for each construct in melanoma cell lines. B) Schematic diagram as described above, and activity of the p1200ALCAMLuc construct containing a wild-type (filled circle) or mutant (open circle) consensus binding sequence for NF-κB in the ALCAM promoter.

Preliminary studies showed that DNA sequences upstream of -650 of the ALCAM gene drove expression of a promoter-less luciferase gene in a wide variety of cell types, and that this activity required an intact Sp1 element at -550 (data not shown). Transient activities of constructs p650, p800, p1000 and p1200 ALCAMLuc were virtually identical. Activity of p1200ALCAMLuc was 2-3-fold higher than of p1000ALCAMLuc (Fig. 3A) in three melanoma cell lines (C8161.9, LOX and Meljuso), which suggested the presence of a positive *cis*-acting element within the sequence between -1200 to -1000. A putative NF-κB sequence was identified at -1140, this motif was mutated using site-directed mutagenesis (GGGGTTGCCC→GGAATTGCCC) to investigate its role in ALCAM promoter activity. This mutation reduced activity of the p1200 construct to the level found in the p1000 construct (Fig. 3B). We presumed that NF-κB bound to this cognate element in the ALCAM promoter, and tested this hypothesis using a complement of *in vitro *and *in vivo *DNA-protein assays.

**Figure 4:**
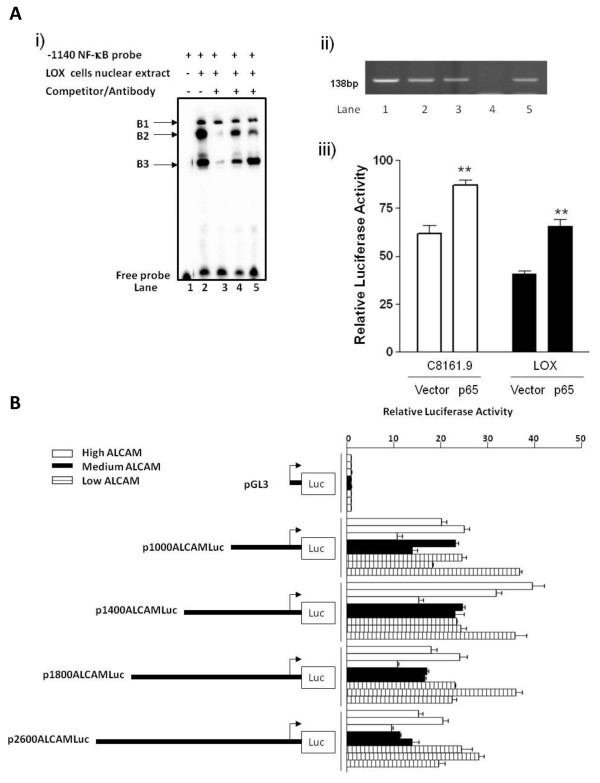
**NF-κB binding to the ALCAM promoter and activity of the ALCAM promoter in breast cancer cell lines**. A) p65 binding and trans-activation of the ALCAM promoter. (i) EMSA showing mobility of the -1140 NF-κB probe in the absence of nuclear extract (lane 1). Complexes B1, B2 and B3 form in the presence of LOX melanoma cell nuclear extracts (lane 2), subsequent lanes contain unlabelled wild-type NF-κB probe (lane 3), unlabelled mutant NF-κB probe (lane 4) and anti-p65 antibody (lane 5). (ii) ChIP assay showing PCR products for the -1140 ALCAM NF-κB motif and flanking DNA sequence amplified from chromatin of C8161.9 (lane1), LOX (lane 2) and Meljuso (lane3) cells precipitated with anti-p65 antibodies. Absence of PCR product in immunoprecipitation with non-immune IgG (lane 4), lane 5 (input DNA). (iii) Activity of p1200ALCAMLuc in C8161.9 and LOX cells over-expressing p65 or control vector. B) Schematic diagram of ALCAM reporter constructs and histogram showing relative luciferase activity for each in breast cancer cells with high (HCC70, MDA-MB-231, T47D), medium (BT549, CAMA-1) and low (HCC 1500, MCF-7,-SK-BR-3) ALCAM expression.

LOX cell nuclear extracts formed three unique complexes (B1, B2 and B3) of variable intensities with a biotin-labeled -1140 ALCAM NF-κB probe (Fig. 4A (i), lane 2). Competition with a 50-fold molar excess of unlabeled wild-type probe abolished assembly of complex B2 and B3 (lane 3), while an unlabeled mutant probe with a 2-bp substitution of the NF-κB consensus sequence (GGGGTTGCCC→GGAATTGCCC) had negligible or reduced impact on B2 and B3 complex formation (lane 4). Anti-p65 antibody markedly reduced the intensity of complex B2 (lane 5), and B3 to a moderate extent. CHIP analysis using anti-p65 antibodies confirmed occupancy of p65 on the endogenous ALCAM promoter in all three melanoma cell lines (Fig. 4A (ii), lanes 1-3). Collectively, these data show that protein complexes containing the p65 NF-κB subunit bind to a cognate sequence at or around the -1140 element on the human ALCAM promoter. To validate these binding experiments, a DNA construct expressing NF-κB (pCMV-65) was used in co-*trans*-activation assays, and this showed that co-transfection of 500 ng p1200 ALCAMLuc with 100 ng pCMV or pCMV-65 plasmid DNA significantly increased ALCAM promoter activity only in conjunction with the p65 expression vector (Fig. 4A (iii). We concluded from these data that NF-κB activates the human ALCAM promoter.

Next, we assessed the activity of various ALCAM promoter constructs truncated at -650, -800, -1000 and -1200 in breast cancer cells, and discovered no appreciable activity, even though these same cells supported high level activity of a CMV promoter driving a β-gal reporter gene (data not shown). We presumed this was likely due to the presence of negative regulatory elements and therefore assayed additional DNA constructs truncated at -1400, -1800 and -2600. Activity of these larger promoter fragments remained relatively low in breast cancer cells irrespective of the level of ALCAM expression (Fig. 4B and data not shown).

### DNA methylation silences ALCAM expression in tumor cells

**Figure 5:**
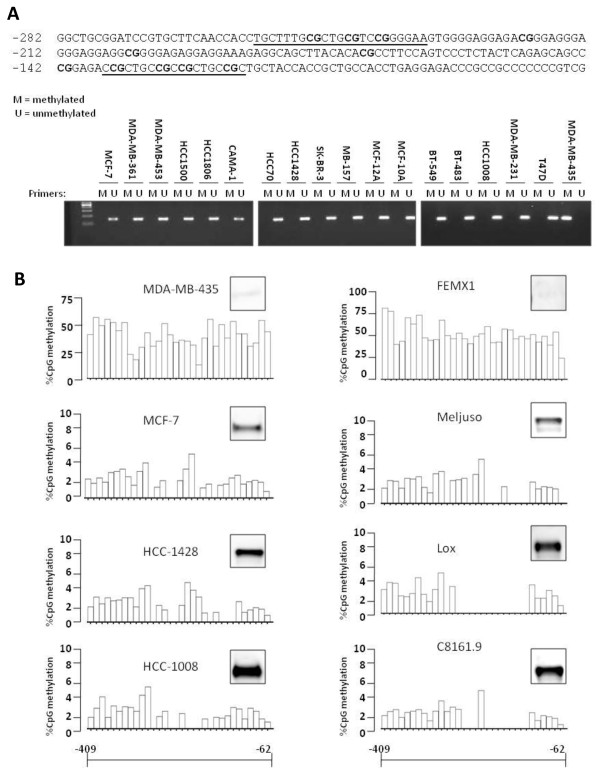
**Promoter methylation regulates ALCAM expression in tumor cells**. A) Sequence of the proximal ALCAM promoter showing multiple CpG islands (in bold) and methylation-specific primer targets (underlined). Ethidium bromide stained agarose gel showing PCR products for unmethylated (U) and methylated (M) genomic DNA. B) CpG methylation profile of the ALCAM promoter in the interval -62 and -409 relative to the cap site as determined by pyrosequencing for the indicated breast (MDA-MB-435, MCF-7, HCC-1428, HCC-1008) and melanoma (FEMX-1, MelJuso, LOX, C8161.9) tumor cells. ALCAM protein detected by western blot analysis is shown for each cell line. Note that the maximum value on the %CpG methylatyion axis (y) for the ALCAM-negative tumor cells MDA-MB-435 and FEMX1 are 75% and 100% respectively, and 10% for cells with endogenous ALCAM expression.

Results from our reporter gene assays highlighted epigenetic modification as the basis for the lack of ALCAM expression in the MDA-MB-435 cell line. This idea was consistent with our sequencing data showing that the ALCAM promoter is GC-rich, and regulated by Sp1 (data not shown). To investigate this idea, genomic DNA from a large panel of breast cancer cells was modified by treatment with sodium bisulfite, and amplified using primers that discriminate methylated and unmethylated DNA (Fig. 5A). Unmethylated (U) DNA was specifically amplified in all breast cancer cell lines with detectable ALCAM expression, but not in the MDA-MB-435 cell line, which lacks endogenous ALCAM (Fig. 5A). Conversely, PCR product of methylated (M) DNA was present only in reactions using MDA-MB-435 DNA (Fig. 5A). Sequencing of bisulfite-modified DNA revealed that virtually all CpG islands in the proximal ALCAM promoter were methylated in MDA-MB-435 tumor cells, with percentage methylation ranging from 15% to 60% (Fig. 5B). A similarly high degree of methylation was found in the FEMX-1 melanoma cell line (up to 75% of specific CpG residues), which also lacks ALCAM expression while all other tumor cells with appreciable ALCAM expression had a negligible (0-5%) level of DNA methylation (Fig. 5B).

**Figure 6:**
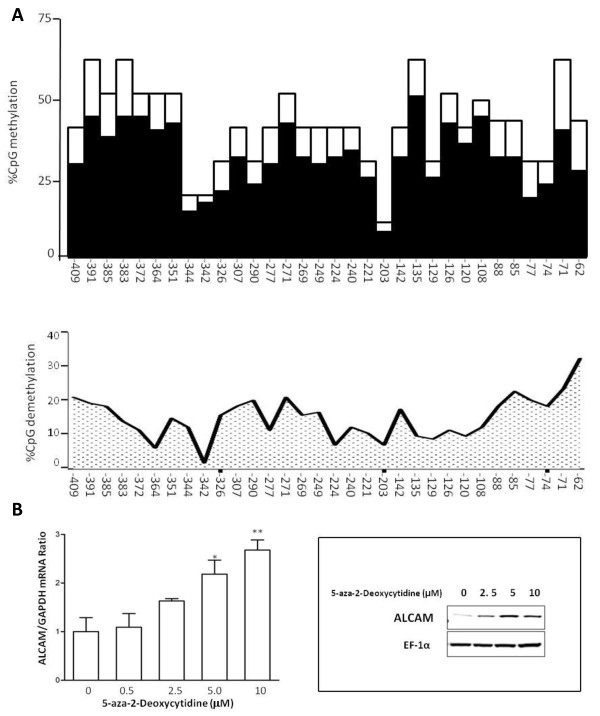
**5-aza-deoxydytidine activates ALCAM expression in tumor cells**. A) top panel; Histogram showing baseline methylation at each CpG sites in the ALCAM promoter in MDA-MB-435 cells (blank columns) and the percent methylation following treatment with 5 μM 5-aza-deoxycytidine (filled columns). Lower panel; Profile of DNA demethylation across the proximal ALCAM promoter achieved by treating MDA-MB-435 cells with 5 μM 5-aza-deoxycytidine. B) Dose-dependent re-activation of ALCAM mRNA and protein expression in MDA-MB-435 cells treated with a concentration range (0-10 μM) of 5-aza-deoxycytidine.

To directly confirm that CpG methylation silences the ALCAM gene, MDA-MB-435 cells were treated with 5-aza-2-deoxycytidine which reduced methylation at each CpG site by 3 to 37% at concentrations of 5 μM and 10 μM of the drug (Fig. 6A and data not shown). This modest degree of demethylation was accompanied by significantly increased ALCAM expression at both mRNA and protein levels in MDA-MB-435 cells (Fig. 6B). Collectively, these data identify CpG methylation of the ALCAM promoter as one mechanism for controlling ALCAM gene expression.

### ALCAM clusters tumor cells perfusing through an isolated rat lung

**Figure 7:**
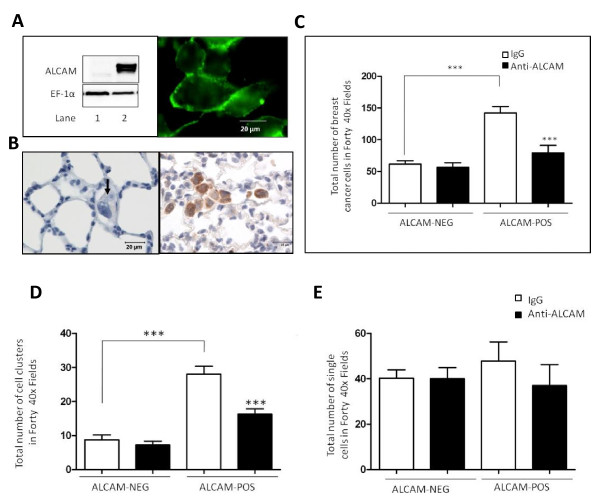
**ALCAM clusters tumor cells circulating through the lung**. A) Ectopic expression of ALCAM in MDA-MB-435 tumor cells. *Left panel*; Western blot analysis of ALCAM in MDA-MB-435 clones transfected with an empty vector (lane 1) or, an ALCAM-GFP vector (lane 2). Protein loading was verified by probing the same western blot filter for the house keeping protein EF1-α. *Right panel*; Monolayer of MDA-MB-435-ALCAM-GFP cells were examined by live-cell microscopy to reveal expression of ALCAM at sites of cell-cell contact. B) Medium power image show virtual absence of control MDA-MB-435 cells, except for a single cell (arrow) while the ALCAM-expressing MDA-MB-435 clones form clusters (brown stain) after 90 minute perfusion in rat lungs. C) Number of tumor cells retained in rat lungs in experiments using ALCAM-positive (n = 7), or ALCAM-negative MDA-MB-435 cells (n = 4) pre-treated with non-immune IgG or monoclonal anti-ALCAM antibody. D) Number of tumor cell clusters retained in rat lungs in experiments using ALCAM-positive and ALCAM-negative MDA-MB-435 cells pre-treated with non-immune IgG (n = 7) or monoclonal anti-ALCAM antibody (n = 7). E) Number of single tumor cells retained in rat lungs in experiments using ALCAM-positive and ALCAM-negative MDA-MB-435 cells pre-treated with non-immune IgG (n = 7) or monoclonal anti-ALCAM antibody (n = 7).

The isolated ventilated perfused rat lung system has previously been used to investigate the fate of circulating tumor cells, and was employed here to test the influence ALCAM has on these cells. Stable transfection of an ALCAM-GFP construct conferred high level ALCAM expression in MDA-MB-435 cells, and importantly localized ALCAM to sites of cell-cell contact in confluent cultures (Fig. 7A). Perfusion of this clone into rat lungs caused congestion of tumor cells in the pulmonary vasculature, while relatively few cells were retained in experiments using a control clone expressing an empty vector (Fig. 7B). Quantitative analysis revealed more than 2-fold increase in the number of the ALCAM-positive MDA-MB-435 cells retained in the rat lung (Fig. 7C), compared to the ALCAM-negative variant. Pre-treating ALCAM-positive MDA-MB-435 cells with anti-ALCAM antibodies prior to perfusion significantly reduced the number of tumor cells in the rat lung (Fig. 7C). Additional control experiments showed that anti-ALCAM antibodies did not alter the total number of ALCAM-negative MDA-MB-435 tumor cells retained in the rat lung.

### Anti-ALCAM antibody inhibits clustering of perfusing tumor cells

**Figure 8:**
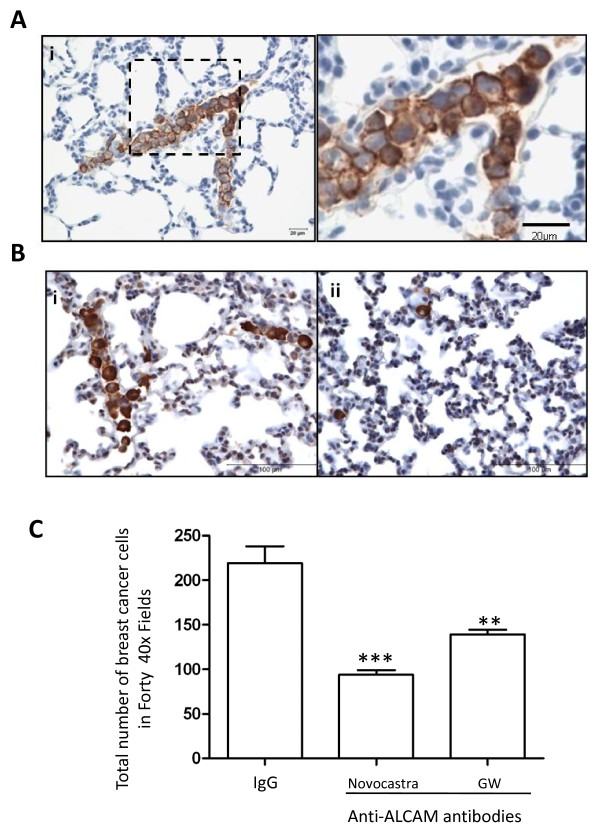
**Anti-ALCAM antibodies reduce clustering of tumor cells circulating through the lung**. A) (i) Medium power image of a rat lung section show MDA-MB-231 tumor cells logged in a medium-size vessel, ii) magnified section of the area enclosed by the dotted box in (i). B) Low-power image of rat lung section show MDA-MB-231 tumor cells in experiments with non-immune IgG (i) or anti-ALCAM antibody (ii). C) Number of tumor cells retained in isolated perfused rat lungs following incubations with non-immune IgG (n = 11) and Novocastra (n = 4) and GW (n = 7) monoclonal anti-ALCAM antibodies.

Next, we focused on the mechanisms of retention of tumor cells in the lung by counting the number of intravascular tumor cell clusters and single tumor cells adherent to endothelial walls. ALCAM expression increased by 3-fold the number of MDA-MB-435 tumor cell clusters, while anti-ALCAM antibody reduced this number significantly (Fig. 7D). On the contrary, ALCAM expression had no significant impact on the number of single MDA-MB-435 tumor cells adhering to the endothelial wall, and anti-ALCAM antibody did not influence this number (Fig. 7E). These data indicate that the presence of ALCAM enhances retention of tumor cells in the pulmonary vasculature primarily as clusters of cells tightly adherent to each other, and not as individual cells adherent to the endothelium. Recent findings indicate that the parental MDA-MB-435 cell line is melonocytic in origin and may therefore exhibit adhesive behaviors atypical of breast cancer cells. To address this concern and also confirm our findings in cells endogenously expressing ALCAM, perfusion experiments were performed using MDA-MB-231. These highly-expressing ALCAM tumor cells formed several clusters while perfusing the pulmonary vasculature of the rat lung (Fig. 8A), two function blocking ALCAM antibodies consistently reduced significantly the total number retained in the rat lung, while IgG had no impact (Fig. 8B, C).

## Discussion

Altered expression of the cell adhesion molecule ALCAM is associated with progression, metastasis and response to therapy in multiple cancers, yet specific DNA elements that regulate ALCAM promoter activity have not previously been defined. We found a consensus NF-κB element at -1140 and site-directed mutagenesis of this element significantly reduced ALCAM promoter activity. Over-expression of p65 NF-κB increased ALCAM promoter activity, while p65 occupied the cognate motif on the endogenous ALCAM promoter in melanoma cells lines (Fig. 4A ii). These data strongly suggests that ALCAM is a target of the NF-κB pathway. Our data in melanoma cells is consistent with previous reports showing that transformation of avian lymphoma B-cells with *v-rel *induces ectopic expression of ALCAM [32]. Mutations of cell cycle genes activate NF-κB, which in turn is directly responsible for increasing expression of several target genes involved in melanogensis [33-36]. Given the marked elevation of expression of ALCAM in vertically growing primary melanoma tumors, it is likely that the ALCAM gene locus is a downstream target of NF-κB in melanoma. Our reporter gene analysis did not reveal *cis*-active elements in the ALCAM promoter that suppress ALCAM expression in breast cancer cell lines. However, the human ALCAM gene locus is relatively large [37] and may contain negative regulatory elements far upstream of the transcription start site, where they may influence gene expression, via long-range DNA looping.

Epigenetic modification of DNA is a well established mechanism for suppressing genes, and we discovered that the proximal ALCAM promoter is endowed with multiple CpG islands, which are targets for DNA methylation (Fig. 5A). Sequencing revealed that virtually all CpG islands in the ALCAM promoter are methylated in tumor cells lacking ALCAM expression. The plasticity of this modification, and its inherent linkage to gene expression was confirmed in experiments with 5-aza-deoxycytidine (Fig. 6A, B). Additional correlative data in both breast cancer and melanoma cell lines showed direct relationship between the level of DNA methylation of the ALCAM promoter and ALCAM protein level (Fig. 5B). 5-Aza-deoxycytidine is currently in clinical use, and may be indicated to boost ALCAM expression in breast cancer [38], however, it also alters expression of a large number of genes [39,40]. Moreover, our data suggests this drug alone maybe insufficient to fully restore ALCAM expression in tumors with severely repressed ALCAM expression. The level of ALCAM expression is variable in different tumor types. This heterogeneity can be resolved by the unique tissue origins of different tumors, although variable expressions have been described at different stages of tumor development in the same type of malignancies. With respect to breast cancer, multiple studies have examined ALCAM expression at the transcript and protein levels, using a variety of methods. There is an emerging consensus that low level ALCAM is a bad prognostic marker in breast cancer [23,24,27,41-44]. In the largest, and most recent study, Ihnen *et al *found that low ALCAM mRNA was associated with shorter disease free survival and duration of survival in hierarchical cluster analysis involving training and multiple validation cohorts of breast cancer patients [44]. This emerging paradigm is supported by the correlations of high ALCAM mRNA with progesterone and estrogen receptor status, better response and longer overall survival. It is reasonable to presume that down-regulation of ALCAM activates alternative compensatory pathways. Plausible candidates are other ALCAM isoforms, such as soluble ALCAM, which may account for the elevation of serum ALCAM in patients with breast cancer [45]. A similar mechanism may explain the marked increased in cytoplasmic ALCAM in some aggressive breast cancer tissues [26]. While the relationships between these various deregulations of ALCAM expression remain to be verified, they all represent loss of function of ALCAM, which to date has consistently been associated with poor prognosis in breast cancer.

Alteration of adhesion molecule expression is a hallmark of several cancers [46], however the underlying biological mechanism for the deleterious effects of reduced ALCAM in breast cancer is poorly defined. In this study, we examined the impact of ALCAM on the adhesive behavior of tumor cells in the pulmonary vasculature using the isolated rat lung system. Experiments using two function blocking ALCAM antibodies, and genetically-modified MDA-MB-435 clones ectopically expressing ALCAM, revealed that ALCAM promotes homotypic tumor cell adhesion demonstrated by clusters of tumor cells in the pulmonary vasculature. ALCAM is localized at sites of lateral cell contacts in the pulmonary endothelium, and is therefore unlikely to mediate adhesions between circulating tumor cells and the endothelium [47]. Colonization of the lung by tumor cells involves their interaction with the endothelial wall, and subsequent extravasations. The data reported here suggests that ALCAM is likely to slow down this process, by promoting homotypic tumor cell adhesion. This idea is pertinent to the level of ALCAM expression and the metastatic phenotypes of the two tumor cells lines we used in our lung perfusion experiments. The ALCAM-positive MDA-MB-231 cells which formed large cell clusters in the rat lung cannot metastasize to distant sites when injected into the mammary fat pad of athymic nude mice [48,49]. On the contrary, ALCAM-negative MDA-MB-435 efficiently and spontaneously forms distant metastasis under identical experimental conditions [48-51]. Additional mechanistic studies are needed to clearly define the relationship between ALCAM level and the metastatic phenotypes of tumor cells. In this study, we have shown that loss of ALCAM function in MDA-MB-231 and gain of ALCAM function in MDA-MB-435, switches their adhesive phenotypes in the pulmonary vasculature, a process that influences metastasis to the lung [30,31].

## Conclusions

The ALCAM promoter is extensively methylated in tumor cells that lack ALCAM expression. Using genetic and antibody blocking assays we demonstrate that ALCAM enhances homotypic adhesion of tumor cells perfusing through the pulmonary vasculature. Thus, reduced homotypic tumor cell adhesion may explain why low-level ALCAM is a risk factor for metastasis and early death in breast cancer. Therapeutic strategies that re-activate ALCAM expression in breast cancer tumors may slow-down tumor metastasis and improve survival.

## Methods

### Cells

Cells studied included MDA-MB-435, originally classified as of breast cancer origin, but recently shown to posses melanocytic lineage, fifteen breast cancer (BT549, BT483, MDA-MB-231, HCC70, HCC1428, HCC1806, MDA-MB-453, MCF-7, CAMA-1, MB-157, MDA-MB-361, HCC1500, HCC1008, T47D and SK-BR-3) and two normal epithelial breast cell lines (MCF-10A and MCF-12A) purchased from American Type Culture Collection (ATCC, Rockville, MD). In addition, four melanoma cell lines (FEMX-I, LOX, MelJuso, C8161.9) were studied. All cell lines were cultured using media conditions recommended by the commercial or academic suppliers. Cells were incubated at 37°C in a humidified chamber with 5% CO_2 _(except for MDA-MB-361, which was cultured in room air). MDA-MB-435 cells (2 × 10^5 ^per 35-mm well) were plated overnight, and then treated with a concentration range (0-10 μM) of 5-aza-2-deoxycytidine (EMD, Madison, WI). Cells were replenished with fresh medium containing 5-aza-2-deoxycytidine every 48 hours for six days, and harvested for analysis.

### Western Blots

Cell lysates (20 μg) were resolved on a 10% SDS-PAGE. Proteins were transferred to PVDF membrane and probed with antibodies (ALCAM/CD166, Novocastra Laboratories) and secondary antibody conjugated to horseradish peroxidase and detected using chemiluminescence (Pierce Biotechnology; Rockford, IL).

### Quantitative RT-PCR and 5' RACE

Total RNA was extracted using RNeasy Mini Kit (Qiagen, Valencia, CA) and converted to cDNA from 2 μg total RNA by SuperScript RT II (Invitrogen, Carlsbad, CA). Quantitative RT-PCR was performed using an ABI StepOnePlus analyzer (Applied Biosystems, Foster City, CA) with SYBR Green master mixture containing primers for ALCAM (NM_001627) or GAPDH (Additional file 1: Table S1). The start of RNA synthesis was identified using the rapid amplification of cDNA ends (RACE) approach (5'RACE, Invitrogen). Briefly, ALCAM cDNA was synthesized from 3 μg RNA using SuperScript RT II and ALCAM-specific primer AL1 (Additional file 1: Table S1), and purified using SNAP column (Invitrogen), tailed with TdT and amplified by PCR using abridged anchor primer (AAP) and ALCAM-specific primer AL2. PCR product was amplified using a nested ALCAM-specific primer AL3 and universal amplification primer (UAP), cloned into a T-vector and sequenced.

### DNA constructs and stable cell lines

Human genomic DNA was amplified by PCR using a common reverse primer and various forward primers truncated at -2600, -1800, -1400, -1200, -1000, -800, -650, -400 and -200 (Additional file 1: Table S1). PCR products were cloned into a promoter-less luciferase vector (pGL3, Promega, Madison, WI) via *Mlu *I and *Bgl *II, and verified by DNA sequencing. We have previously described construction of a fusion DNA vector expressing ALCAM and enhanced green fluorescent protein (GFP) [47]. ALCAM-GFP and control GFP vector (Applied Vironomics, Fremont, CA) were transfected into log-phase growing MDA-MB-435 cells using lipofectamine 2000 (Invitrogen). Stable lines of MDA-MB-435-ALCAM-GFP and MDA-MB-435-GFP were selected using G418 (500 μg/ml).

### Reporter assays

Cells (8 × 10^4^) were seeded in 24-well tissue culture plates and co-transfected with ALCAM promoter luciferase plasmids (800 ng) and pcDNA3.1/His/LacZ (100 ng) (Invitrogen) plasmid DNA using lipofectamine 2000. Twenty-four hours after transfection cell lysates were prepared and the activities of luciferase (Firefly-Luciferase Reporter Assay System, Promega) and β-galactosidase (Galacto-Star system, Applied Biosystems) determined using the Veritas Luminometer (Turner Biosystems, Sunnyvale, CA). Luciferase activity was normalized to the activity of β-galactosidase, and the relative luciferase activity for test constructs calculated by assigning the normalized luciferase activity of the promoter-less pGL3 construct as 1.0. Minimum of three independent experiments was performed for each reporter each in triplicate.

### Electrophoretic mobility shift assay

*In vitro *protein-DNA interaction was examined using the LightShift Chemiluminescent electrophoretic mobility shift assay (EMSA) kit (Pierce). ALCAM-specific EMSA DNA probes were synthesized, gel purified and biotin labeled (Additional file 1: Table S1). Nuclear extract (4 μg) was combined with biotin-labeled DNA probes in binding buffer containing 50 μg/ml poly(dI-dC). Fifty-fold molar excess of unlabelled DNA probe was added to the binding reaction in competition experiments. Anti-p65 NF-κB antibodies (2 μg) (Santa Cruz) were added to the reaction mixture. Products of the binding reaction were resolved in 6% DNA retardation gel, transferred to a nylon membrane and biotin-labeled complexes detected by chemiluminescence (Fujifilm LAS-1000 imaging system; FujiFilm, Valhalla, NY).

### Chromatin immunoprecipitation assay

Protein-DNA cross-linking was performed by fixing 40 million cells with 1% formaldehyde. Nuclei was sonicated on ice in shearing buffer (ChIP-IT; Active Motif, Carlsbad, CA) to obtain chromatin fragments of 100-1000 base pairs, which were pre-cleared with protein G beads (Salmon sperm DNA/Protein G agarose). Pre-cleared chromatin was incubated with anti-p65 NF-κB antibody (Santa Cruz) or non-immune IgG. Immune complexes were precipitated with protein G beads, and the eluate reversed cross-linked in 190 mM NaCl. DNA was purified and amplified by PCR with specific ALCAM primers (Additional file 1: Table S1).

### DNA methylation

Genomic DNA was extracted using QIAamp DNA Mini Kit (Qiagen) and 1 μg of this sample treated with sodium bisulfite (EZ Methylation Gold kit, Zymo Research, Orange, CA). Bisulfite-modified DNA (1 μl) was amplified in two separate PCR reactions using primers flanking the interval -256 to -118 of the ALCAM promoter and specific for methylated and unmethylated genomic DNA (Additional file 1: Table S1). For bisulfite sequencing biotinylated reverse primers were used in two separate reactions to amplify two distinct and overlapping DNA fragments in the interval -409 to -62. Biotin-labeled single-stranded PCR products were isolated and pyrosequenced (PSQ™96HS System EpigenDx Biotage, Kungsgatan, Sweden). Methylation status of each CpG site was analyzed individually as T/C SNP using QCpG software (Biotage, Kungsgatan, Sweden).

### Isolated perfused lungs

The isolated perfused ventilated rat lung was prepared as we have described previously [52,53]. Adult male Sprague-Dawley rats were anesthetized with pentobarbital sodium (60 mg/kg ip), and a catheter was inserted into the trachea, and the lungs were mechanically ventilated. A median sternotomy was performed, and then heparin (60 units) was administered via the left ventricle and allowed to circulate for 3 min. Catheters were placed and secured around the pulmonary artery and the left atrium. Rat lungs were perfused with Earle's balanced salt solution and 4% bovine serum albumin containing tumor cells at a density of 40,000 cells per ml. In some experiments, tumor cells (2 × 10^6^) were incubated for 1 hour with anti-ALCAM (1:20 Novocastra or 1:20 GW, GenWay Bioetech) or non-immune IgG(control) prior to perfusion. Tumor cells were perfused for 90 minutes followed by perfusion with Earle's balanced salt solution without tumor cells for 5 minutes.

### Immunostaining

Breast cancer cells were fixed with methanol and blocked with 3% normal goat serum, followed by staining with 1/100 dilution of anti-ALCAM (Novocastra Laboratories Ltd), and Alexflour 594 goat anti-rabbit IgG (Molecular Probes, Eugene, Oregon). Nucleus was stained with DAPI (Molecular Probe). Cells were examined by epifluorescence (Nikon TE2000, Nikon Instruments Inc., Melville, NY). Lung tissue was fixed in 10% formalin or 4% paraformaldehyde, processed, embedded in paraffin, and sectioned (4-5 microns). Sections were stained with hematoxylin & eosin (H&E), anti-GFP (1:250; Molecular Probes, Inc., Eugene, OR), or anti-ALCAM (1:40; Novocastra Laboratories Ltd). Tumor cells in twenty 40× fields were counted on each section/slide. A total of forty lung fields (40×) per rat lung were analyzed by light microscopy to assess for tumor cells. The number of tumor cells and presence of single cells or cell aggregates were recorded. Sections were photographed with a Nikon E600 light microscope with digital imaging (Nikon Instruments Inc., Melville, NY).

### Statistics

The data are reported as the means ± SE for at least three independent experiments. Data was graphed and analyzed using Prism software (GraphPad Software). Statistical analysis of the raw data was performed by two tailed *t *tests. Differences were considered significant if *p *value were < 0.05 (*), <0.01 (**) and <0.001 (***).

## List of Abbreviations

ALCAM: activated leukocyte cell adhesion molecule; RACE: rapid amplification of cDNA ends; UAP: Universal Amplification Primer; AAP: Abridged Amplification Primer.

## Competing interests

The authors declare that they have no competing interests.

## Authors' contributions

JAK participated in the design of the studies and performed experiments. FT performed experiments and prepared the manuscript. FM, ZC, SC, HC, DA and LAS performed experiments. SFOA designed the study, analyzed data and wrote the manuscript. All authors read and approved the final manuscript.

## Supplementary Material

Additional file 1 **Sequence of Primers and DNA probes**. Table S1 contains PCR primers, EMSA probe sequence and bisulfite sequencing primers. Click here for file
