# Supplementary material for: Mechanisms of transcriptional regulation and prognostic significance of activated leukocyte cell adhesion molecule in cancer
Source: Mol Cancer. 2010 Oct 7;9:266. doi: 10.1186/1476-4598-9-266 (PMC2958981; doi:10.1186/1476-4598-9-266)
Supplement: Additional file 1 — Sequence of Primers and DNA probes. Table S1 contains PCR primers, EMSA probe sequence and bisulfite sequencing primers. [file 1476-4598-9-266-S1.DOC]

**Table S1. Sequence of Primers and DNA Probes**

| **RT-PCR** | *Forward* | *Reverse* |
| --- | --- | --- |
| ALCAM  GAPDH | 5’-AGTCTTCATTATCAGGATGC-3’  5’-ATGGGGAAGGTGAAGGTCGG-3’ | 5’-GGGATCAGTTTTCTTTGTCA-3’  5’-GACGGTGCCATGGAATTTGC-3’ |
| **5’RACE** | *Reverse* |  |
| AL1 | 5’-CATATTCCTCCTCCTTCTTGGTGGGCCACTGACGGAGTCCACG-3’ | |
| AL2 | 5’-AGGGGCAGCGTCTTCTCCCA-3’ | |
| AL3 | 5’-ACCTTCGGTTCTCTGCCGCA-3’ | |
| **ALCAMLuc reporter constructs** | *Forward* | *Reverse* |
|  |  |  |
| -2600 | 5’-ATATGCATGTGTTTGTGTATAAG-3’ |  |
| -1800 | 5’-ATGTAACGATTACTAGTTGG-3’ | |
| -1400 | 5’-AATCACCGCTTAACTCAAAG-3’ | |
| -1200 | 5’-AAATCACCGCTTAACTCAAAG-3’ | |
| -1000 | 5’-CAGAAAGTGTTAGTCCCAGG-3’ | |
| -650 | 5’-CCGCGCTTCAACCACCTGCT-3’ |  |
| -400 | 5’-CCGCCTCCTGCGAGTCCTTC-3’ |  |
| -200 | 5’-GTTGACCGGGAGGGAGGAGG-3’ |  |
| -60 |  | 5’-CCTCCTCCTTCTTGGTGG-3’ |
| **EMSA probes** | *Sense strand* |  |
| -1140 NF-κB wt | 5’-GTACAGGGGTTGCCCCTGCC-3’ | |
| -1140 NF-κB mt | 5’-GTACAGGAATTGCCCCTGCC-3’ |  |
| **ChIP assay** | *Forward* | *Reverse* |
| NF-κB | 5’-TGAGGGCAGTGGTGGGGGTG-3’ | 5’-GTGGCAAAGGTGGAAACTTG-3’ |
| **Methylation-specific PCR** | *Forward* | *Reverse* |
| ALCAM-M | 5’- TGTTTTGCGTTGCGTTCGGGGA -3’ | 5’-ACAACAACGACGACAACGATCT-3’ |
| ALCAM-U | 5’- TGTTTTGTGTTGTGTTTGGGGA -3’ | *5’-* ACAACAACAACAACAACAATCT-3’ |
| **Bisulfite sequencing** | *Forward* | *Reverse* |
| PCR-1-Primer | 5’-GAGTTGTAATATTATAGAAAGTGTTA-3’ | 5’-TATAAACTACCTCTTTCCTCCTCTC -3’ |
| Sequencing-primer | 5’- GGTTTTTGTGTTTGTTTCTTAT -3’ |  |
|  | 5’- GTTTAGGGTTYGGATTTAGTTCTGTA -3’ |  |
| PCR-2-Primer | 5’-GGAGAGGAGGAAAGAGGTAGTTTA-3’ | 5’-AATATACATAAAACCCCAAATACT-3’ |
| Sequencing-primer | 5’-AGTTTTTTTATTTAGAGTAG-3’  5’-ATTATYGTTGTTATTTGAGG-3’ |  |
|  |  |  |
